# Pneumonia of the Winter of 1841–’2

**Published:** 1843-01

**Authors:** 


### PNEUMONIA OF THE WINTER OF 1841–'2.

Many parts of the North, during last winter, were visited by a severe, in some instances malignant, pneumonia. Was it limited to these latitudes, or did it invade the middle and southern? If the latter, we respectfully ask for detailed information on its symptoms, treatment, anatomical lesions, and sequelæ, promising that the general results of the whole shall in due time be given to our readers.

